# Tooth morphology of deep‐water catsharks of the genus *Apristurus* (Chondrichthyes: Pentanchidae) in the North Atlantic Ocean

**DOI:** 10.1111/jfb.70537

**Published:** 2026-06-14

**Authors:** Jesco Seifert, Daniel M. Moore

**Affiliations:** ^1^ Museum of Natural History Chemnitz Chemnitz Germany; ^2^ Centre for Ecology and Conservation, Faculty of Environment, Sustainability and Economy University of Exeter Exeter UK

**Keywords:** demon catsharks, dental morphology, Elasmobranchii, ghost catsharks, jaws

## Abstract

The deep‐water catshark family (Pentanchidae) is the most species‐rich family among extant shark lineages. Within this family, the genus *Apristurus* is the largest, comprising small, deep‐sea species characterised by elongated bodies and dorso‐ventrally compressed snouts. Five *Apristurus* species are currently recognised from the North Atlantic, including waters surrounding Ireland and the United Kingdom (*A. aphyodes*, *A. laurussonii*, *A. manis*, *A. melanoasper* and *A. microps*). However, their biology remains poorly understood owing to their occurrence at considerable depths and their close morphological similarity. This study presents the first comprehensive comparative analysis of tooth morphology for *Apristurus* species recorded in the region. The results reveal pronounced sexual dimorphism in *A. aphyodes* and provide evidence supporting ontogenetic changes in tooth morphology in *A. melanoasper* and *A. microps*. Furthermore, distinctive morphological characteristics are identified and described for each species using scanning electron microscopy of the dentition. These findings establish novel diagnostic criteria that enhance the reliability of species identification and provide a robust framework for future taxonomic and ecological research.

## 1 INTRODUCTION

The genus *Apristurus* comprises a diverse group of deep‐sea catsharks within the family Pentanchidae and represents one of the most species‐rich yet least well‐resolved lineages of elasmobranchs. Currently, the genus includes over 40 described extant species, with additional putative and extinct taxa recognised. *Apristurus* exhibit a circumglobal distribution across all deep‐sea environments except the polar regions and the Mediterranean Sea, and typically occurs at depths of approximately 200–2500 m. Limited dietary studies suggest extant species generally function as generalist mesopredators (Ebert et al., 1996; Sólmundsson et al., 2025). *Apristurus* species are characterised by elongated, dorso‐ventrally compressed bodies; broad, spatulate snouts; conspicuous patterns of ampullary pores; multicuspid dentition; and distinctive labial furrow configurations, many of which constitute important taxonomic characters (Springer, 1979). Despite these shared traits, species within the genus are often morphologically similar, frequently known from limited material, and differentiated by only subtle morphological characters. This combination has contributed to persistent taxonomic uncertainty within *Apristurus* (Nakaya et al., 2008). However, there is increasing consensus on the genus being subdivided into three morphotypes or ‘species groups’ (Sato, 2000). These species groups are established as the long‐snouted *longicephalus* group (not represented in the north Atlantic), and the short‐snouted species which are divided into two further groups, *spongiceps* and *brunneus* (Nakaya & Sato, 1999).

Documented tooth morphologies and dentition atlases provide valuable reference material, enabling inferences regarding species‐specific feeding strategies and habitat use, as well as serving as important diagnostic tools for species identification (Feichtinger et al., 2025). This is particularly relevant for deep‐water taxa such as *Apristurus*, for which conventional ecological investigation methods are often impractical due to the financial and logistical constraints associated with deep‐sea research (Bouchet & Duarte, 2006). The continued paucity of data on diagnostic characters, including tooth morphology, across a broad range of *Apristurus* species hampers scientific investigation and has led many studies to limit identifications to *Apristurus* sp. (e.g., Etnoyer & Warrenchuk, 2007; Pinte et al., 2020; van Staden et al., 2023). Genus‐level identification is likewise common in the palaeontological literature when describing extinct elasmobranch assemblages based on isolated fossil teeth (Nishimatsu, 2019; Pollerspöck et al., 2023; Underwood & Schlögl, 2012). Consequently, only a single extinct species has been formally described, *Apristurus sereti* (Adnet, 2006).

The absence of detailed descriptions of tooth morphology for *Apristurus* spp. limits not only ecological understanding of extant species but also the ability to reconstruct the composition of extinct deep‐water megafaunal communities. Fossil tooth specimens attributed to *Apristurus* have been reported from several deep‐water deposits, including the middle Eocene (Adnet, 2006), early Miocene (Nishimatsu, 2019; Nishimatsu & Ujihara, 2020; Pollerspöck & Straube, 2017) and Pleistocene (Marsili, 2007). However, *Apristurus* is notably absent from numerous studies of deep‐water fossil assemblages in which its presence might reasonably be expected (e.g., Feichtinger et al., 2025; Herraiz et al., 2023; Martínez‐Pérez et al., 2018). Instead, these assemblages often include teeth attributed to *Scyliorhinus*, a genus whose extant representatives are not known to occur below depths of approximately 800 m. This pattern suggests that the limited availability of appropriate extant comparative material for *Apristurus* may be constraining its accurate identification in deep‐water fossil deposits.

Herein, we document the dentition and tooth morphology of five *Apristurus* species (*A. aphyodes*, *A. laurussonii*, *A. manis*, *A. melanoasper* and *A. microps*) occurring in the northern Atlantic. The inclusion of both male and female specimens across a range of size classes, where available, is intended to provide a valuable reference for future studies of both extant and extinct *Apristurus* species.

## 2 MATERIALS AND METHODS

### 2.1 Sample collection and identification

**FIGURE 1:**
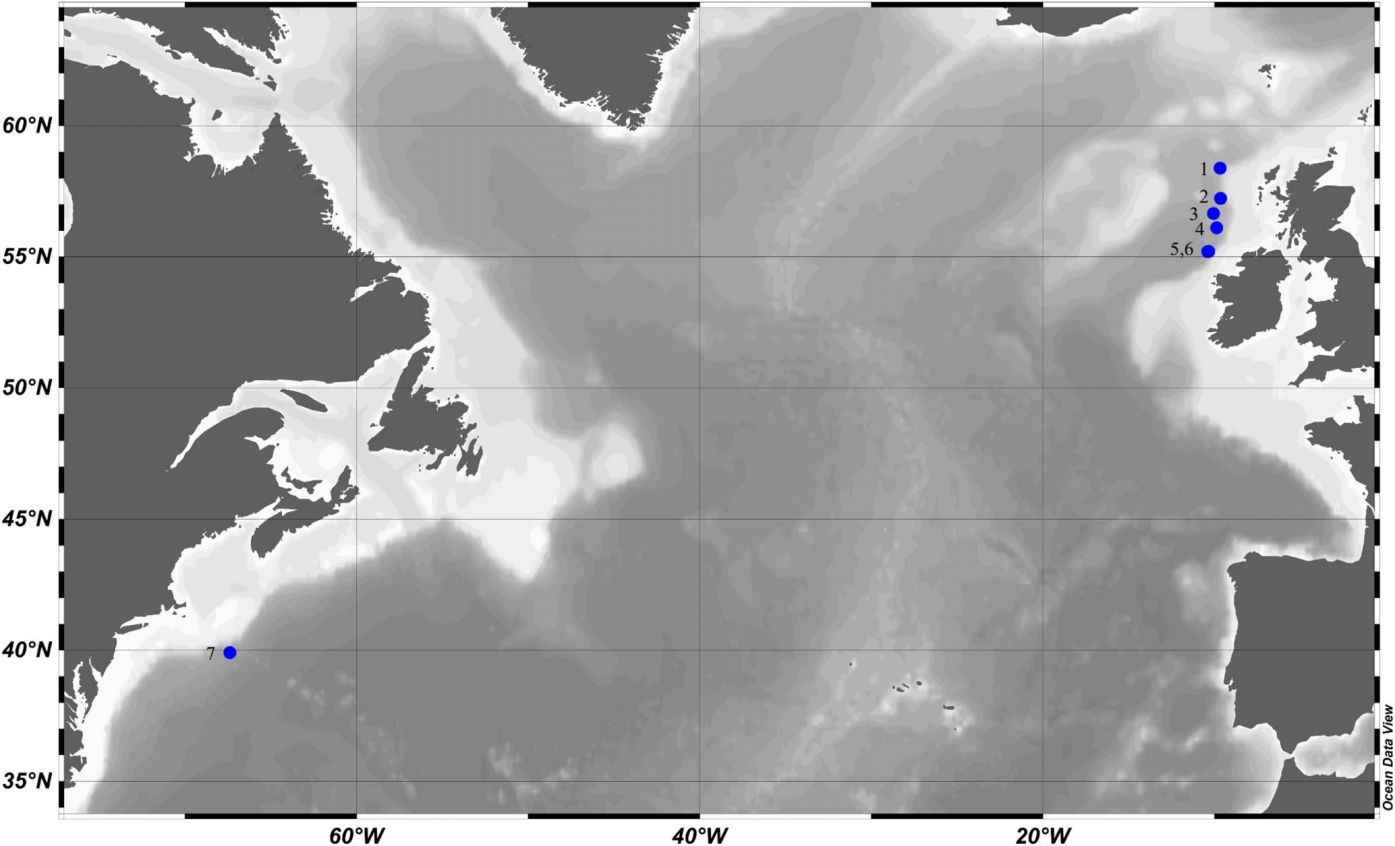
Source of sample material from the north Atlantic. Latitude/Longitude of sample locations are provided in Table 1. *A. aphyodes* specimens were obtained from sample locations 1, 2, 5 and 6. *A. microps* specimens were obtained from sampling location 3. *A. melanoasper* specimens were obtained from sampling location 4 and *A. manis* specimens were obtained from sample location 7.

Specimens of *A. aphyodes*, *A. microps* and *A. melanoasper* were collected using deep‐water trawl within the Rockall Trough (Figure 1) during the Scottish Marine Directorate biennial deep‐water trawl survey aboard the RV *Scotia* in September 2025. Samples were obtained across a range of trawl depths (Table 1), spanning 1510–1998 m. All specimens were transported frozen prior to sample preparation. Species identifications were confirmed based on head morphology and patterns of ampullary pore distribution (McNamara‐Langton et al., n.d.).

**TABLE 1:** Overview of the examined *Apristurus* specimens (N/A = Not available/not known).

Species	Collection number	Sex	Total length (TL) in mm	Weight in g	Catch location	Catch depth in m, catch method	Collection date	Cruise ID	Jaw size (width × height) in mm
*Apristurus aphyodes*	AAP 1063	Male	460	407	55°12′36.0″ N 10°21′14.4″ W (Northeast Atlantic Ocean)	1621, trawl	27 September 2025	1125S	60 × 53
*Apristurus aphyodes*	AAP 4010	Male	538	549	55°12′18.0″ N 10°24′28.8″ W (Northeast Atlantic Ocean)	1866, trawl	25 September 2025	1125S	83 × 60
*Apristurus aphyodes*	AAP 4018	Female	309	109	55°12′18.0″ N 10°24′28.8″ W (Northeast Atlantic Ocean)	1866, trawl	25 September 2025	1125S	37 × 34
*Apristurus aphyodes*	AAP 7234	Male	504	473	57°14′09.6″ N 9°39′03.6″ W (Northeast Atlantic Ocean)	1793, trawl	02 October 2025	1125S	78 × 59
*Apristurus aphyodes*	AAP 7780	Female	465	400	58°23′06.0″ N 9°41′06.0″ W (Northeast Atlantic Ocean)	1510, trawl	05 October 2025	1125S	52 × 40
*Apristurus aphyodes*	AAP 7781	Female	408	208	58°23′06.0″ N 9°41′06.0″ W (Northeast Atlantic Ocean)	1510, trawl	05 October 2025	1125S	60 × 49
*Apristurus aphyodes*	AAP 7782	Female	533	558	58°23′06.0″ N 9°41′06.0″ W (Northeast Atlantic Ocean)	1510, trawl	05 October 2025	1125S	72 × 54
*Apristurus aphyodes*	AAP 7783	Female	394	213	58°23′06.0″ N 9°41′06.0″ W (Northeast Atlantic Ocean)	1510, trawl	05 October 2025	1125S	48 × 40
*Apristurus manis*	DE200304‐001	Male	720	1210	Bear Seamount, ca. 39°55′00.0″ N 67°24′00.0″ W (Northwest Atlantic Ocean)	N/A	20 May 2003	DE200304	109 × 105
*Apristurus manis*	DE200304‐002	N/A	720	N/A	Bear Seamount, ca. 39°55′00.0″ N 67°24′00.0″ W (Northwest Atlantic Ocean)	N/A	20 May 2003	DE200304	95 × 80
*Apristurus manis*	DE200304‐006	N/A	700	N/A	Bear Seamount, ca. 39°55′00.0″ N 67°24′00.0″ W (Northwest Atlantic Ocean)	N/A	20 May 2003	DE200304	95 × 85
*Apristurus melanoasper*	AME 1066	Male	312	95,1	56°07′04.8″ N 9°52′30.0″ W (Northeast Atlantic Ocean)	1813, trawl	28 September 2025	1125S	35 × 31
*Apristurus melanoasper*	AME 1067	Female	688	712	56°07′04.8″ N 9°52′30.0″ W (Northeast Atlantic Ocean)	1813, trawl	28 September 2025	1125S	49 × 44
*Apristurus microps*	AMI 7163	Male	445	347	56°39′36.0″ N 10°04′22.8″ W (Northeast Atlantic Ocean)	1998, trawl	28 September 2025	1125S	68 × 53

Specimens of *A. manis* were collected from Bear Seamount on 20 March 2003 by the National Marine Fisheries Service (NOAA Fisheries) led by John Galbraith and were identified by José I. Castro (Table 1). We note that *A. manis* was placed in the genus *Pentanchus* by Soares and Mathubara (2022), but this change has not been adopted by most authors. To maintain taxonomic stability, we retain its position within *Apristurus*.

The dentition of *A. laurussonii* is described from the published literature.

### 2.2 Sample preparation

The jaws of each specimen were carefully excised using a scalpel. Any adherent tissue was removed, after which the jaws were bleached and cleaned by immersion in a 3% hydrogen peroxide solution for 24 h. Subsequently, they were repositioned and dried, ensuring proper anatomical alignment during the process.

For the preparation of individual tooth images, teeth were dissected from the jaws and immersed in a 5% potassium hydroxide (KOH) solution for 48 h to dissolve adhering soft tissue. They were subsequently treated with a 3% hydrogen peroxide (H_2_O_2_) solution for 24 h. Finally, the teeth were rinsed multiple times with absolute ethanol and air‐dried.

### 2.3 Sample examination

To enable a precise morphological analysis of the dentition, all jaws were subjected to comprehensive measurement and examination. Measurements were obtained using digital callipers with a precision of 0.1 mm. In cases of dried upper jaw perimeters (DUJP) and dried lower jaw perimeters (DLJP), a flexible measuring tape with a measurement accuracy of 0.5 mm was used. The measurement was taken along the jaw arches according to Šanda and De Maddalena (2004), but in contrast to their methodology, only the distance between the last posterior teeth of each jaw quadrant was assessed.

The imaging of the examined jaws and teeth was performed using the following equipment and parameters:

- Sony DSC‐TX30 camera: Standard field of view for jaws (Figures 2, 3, 4 and 5).
- INSKAM Digital Microscope (Model 1600): Magnified view for teeth in situ (Figures 6, 7, 8, 9, 10, 11, 12 and 13).
- Scanning electron microscope (SEM) PhenomXL (Edition XL2; accelerating voltage 5.3 kV; sample pressure 0.10 Pa): High‐resolution, highly magnified images of isolated teeth (Figures 14, 15, 16, 17 and 18).

**FIGURE 2:**
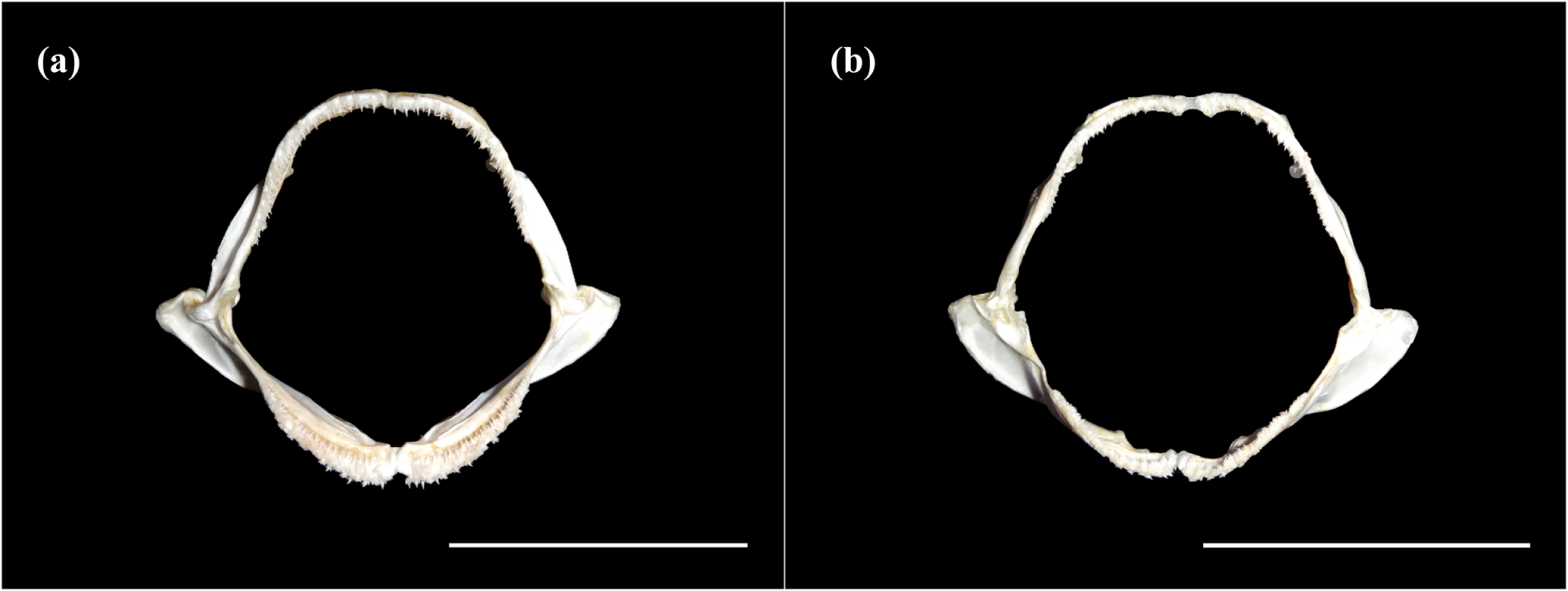
Jaw of *Apristurus aphyodes* specimen AAP 7234 (a; male; 504 mm TL) and AAP 7782 (b; female; 533 mm TL). Scale bar is 50 mm.

**FIGURE 3:**
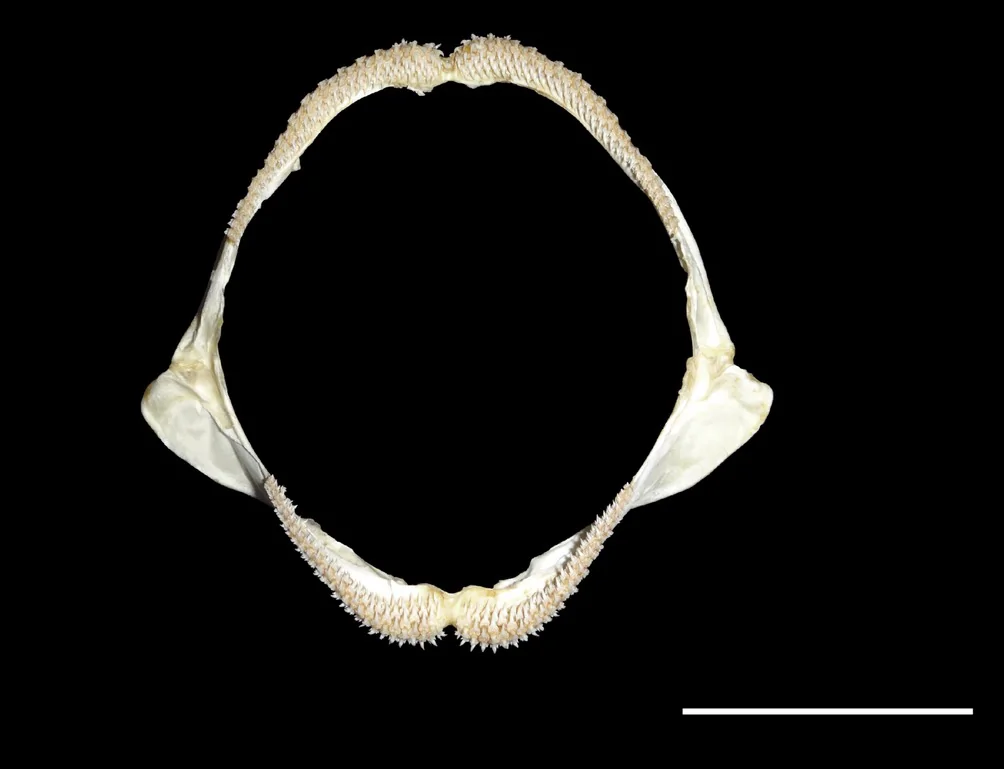
Jaw of *Apristurus manis* specimen DE200304‐001 (male; 720 mm TL). Scale bar is 50 mm.

**FIGURE 4:**
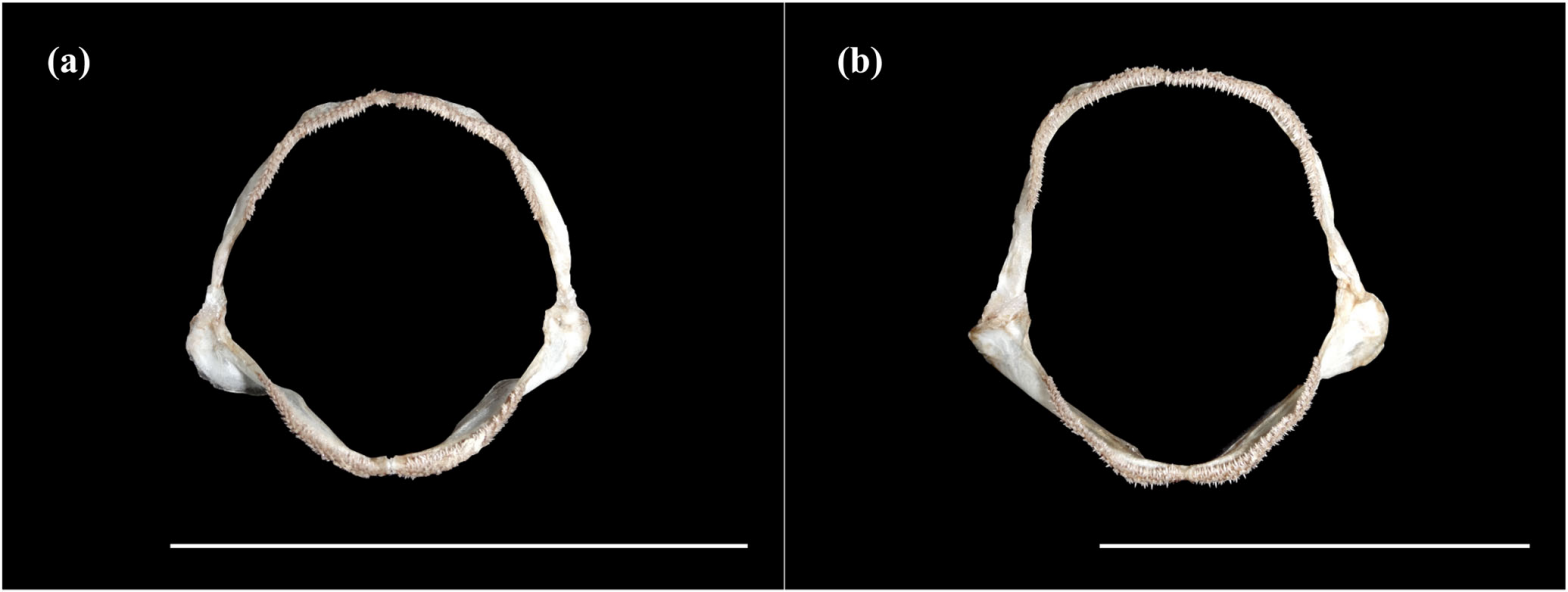
Jaw of *Apristurus melanoasper* specimen AME 1066 (a; male; 312 mm TL) and AME 1067 (b; female; 588 mm TL). Scale bar is 50 mm.

**FIGURE 5:**
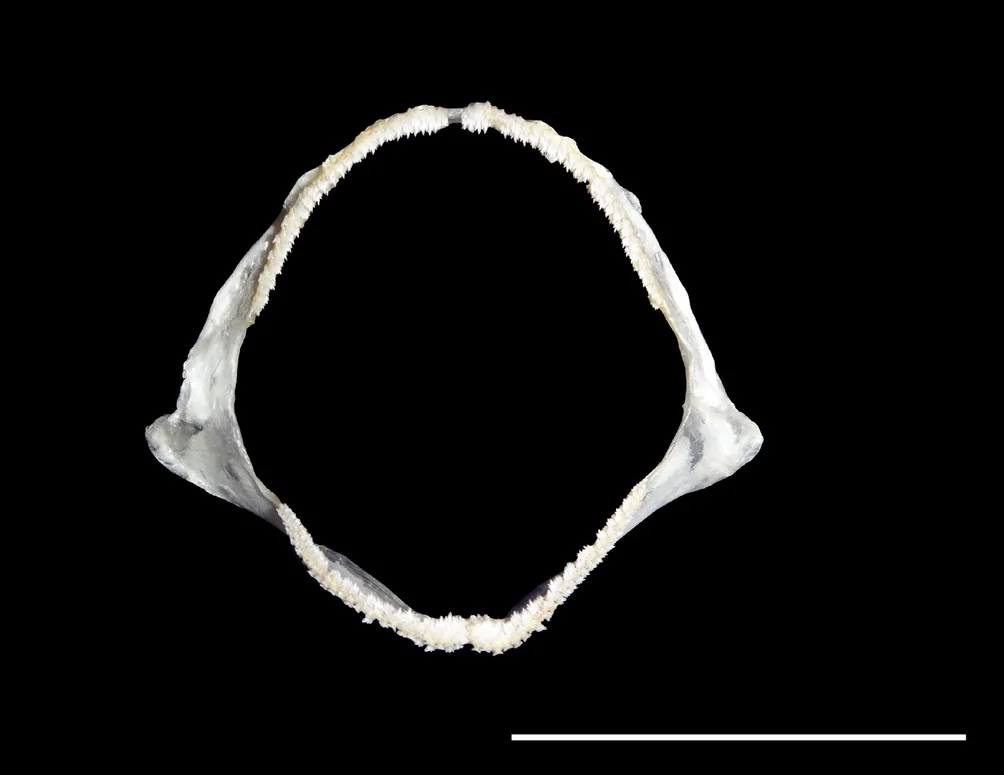
Jaw of *Apristurus microps* specimen AMI 7163 (male; 445 mm TL). Scale bar is 50 mm.

**FIGURE 6:**
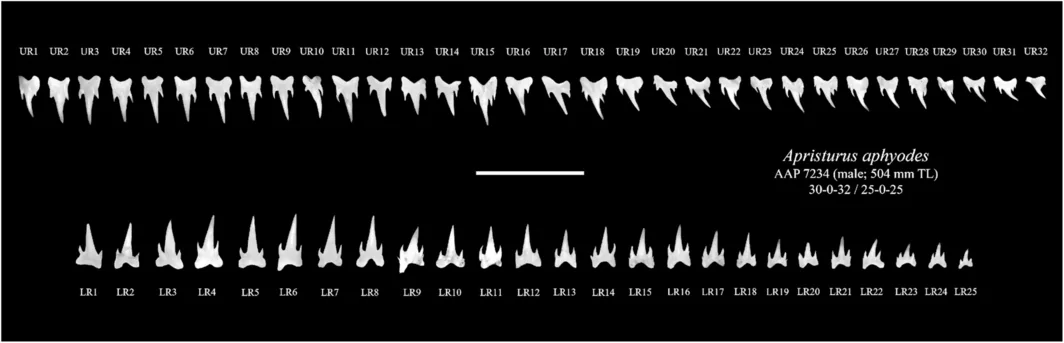
Dentition of the right upper and lower jaw of *Apristurus aphyodes* specimen AAP 7234 (male; 504 mm TL). The image shows the correct position of the teeth from a labial view. Scale bar is 5 mm.

**FIGURE 7:**
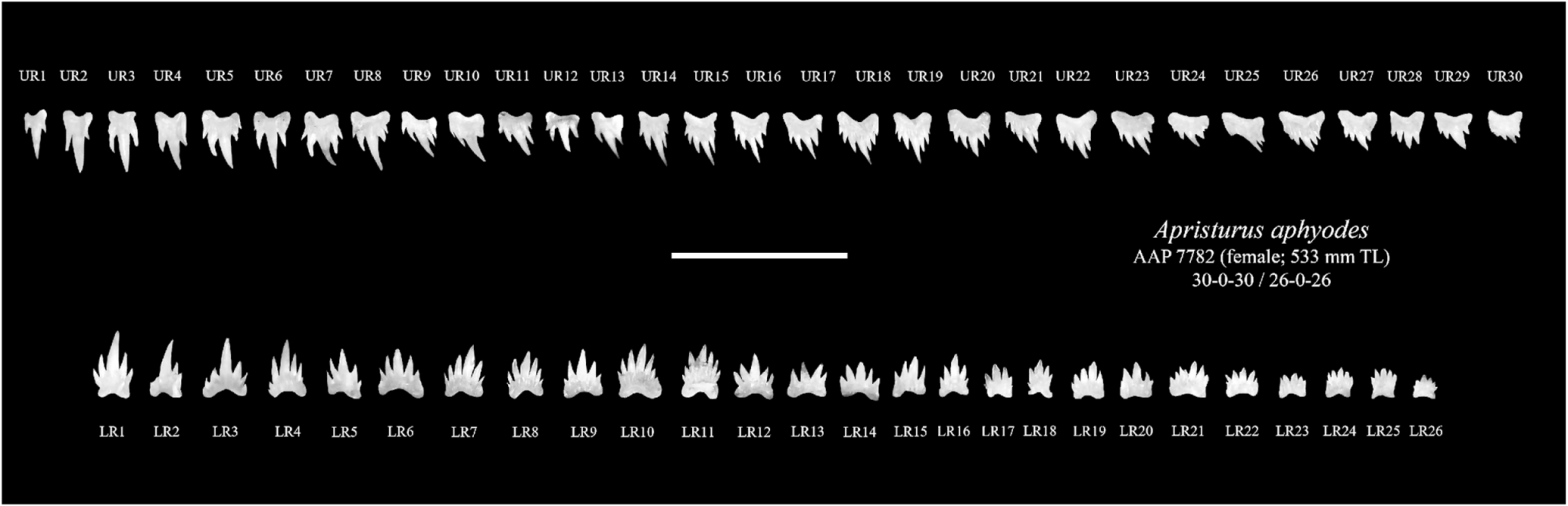
Dentition of the right upper and lower jaw of *Apristurus aphyodes* specimen AAP 7782 (female; 533 mm TL). The image shows the correct position of the teeth from a labial view. Scale bar is 5 mm.

**FIGURE 8:**
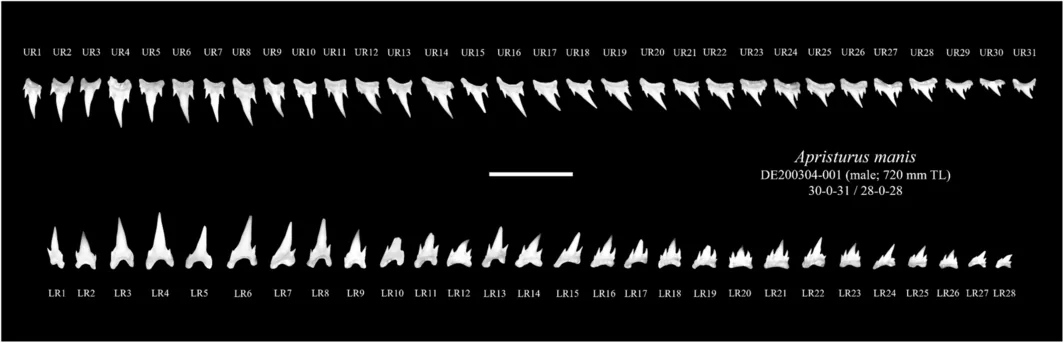
Dentition of the right upper and lower jaw of *Apristurus manis* specimen DE200304‐001 (male; 720 mm TL). The image shows the correct position of the teeth from a labial view. Scale bar is 5 mm.

**FIGURE 9:**
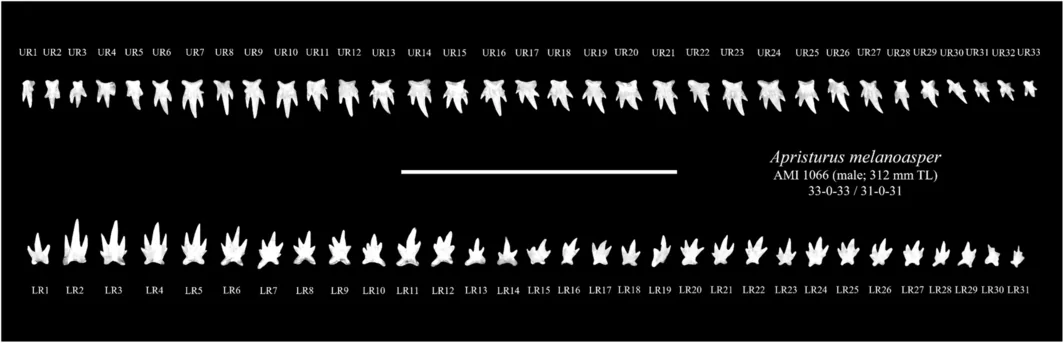
Dentition of the right upper and lower jaw of *Apristurus melanoasper* specimen AME 1066 (male; 312 mm TL). The image shows the correct position of the teeth from a labial view. Scale bar is 5 mm.

**FIGURE 10:**
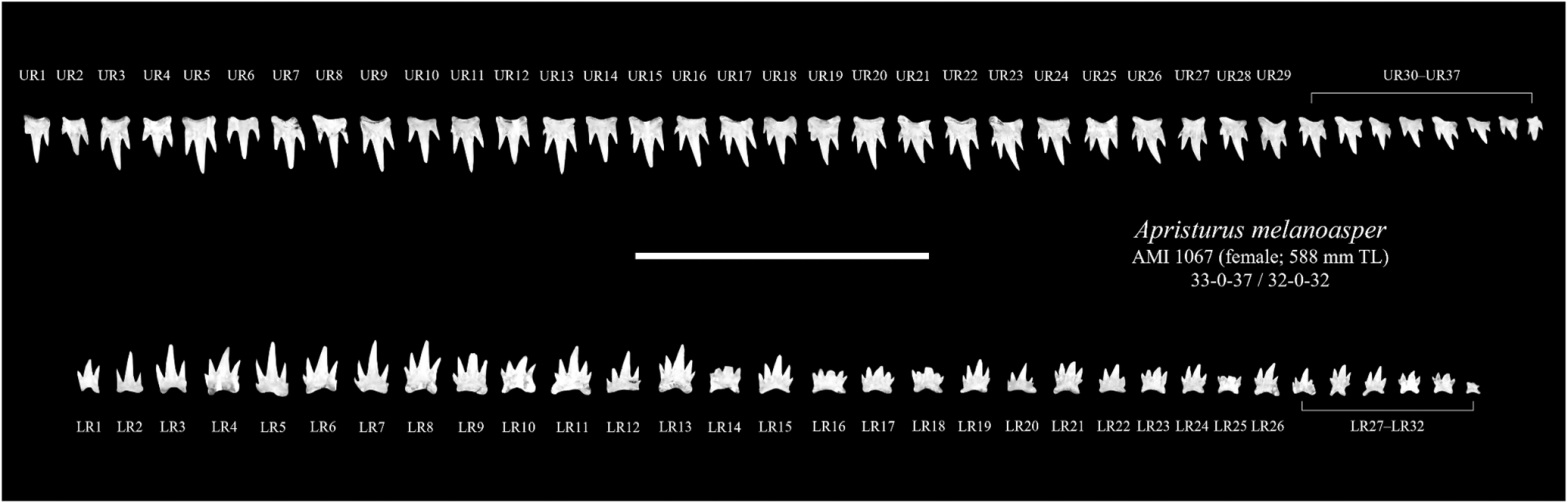
Dentition of the right upper and lower jaw of *Apristurus melanoasper* specimen AME 1067 (female; 588 mm TL). The image shows the correct position of the teeth from a labial view. Scale bar is 5 mm.

**FIGURE 11:**
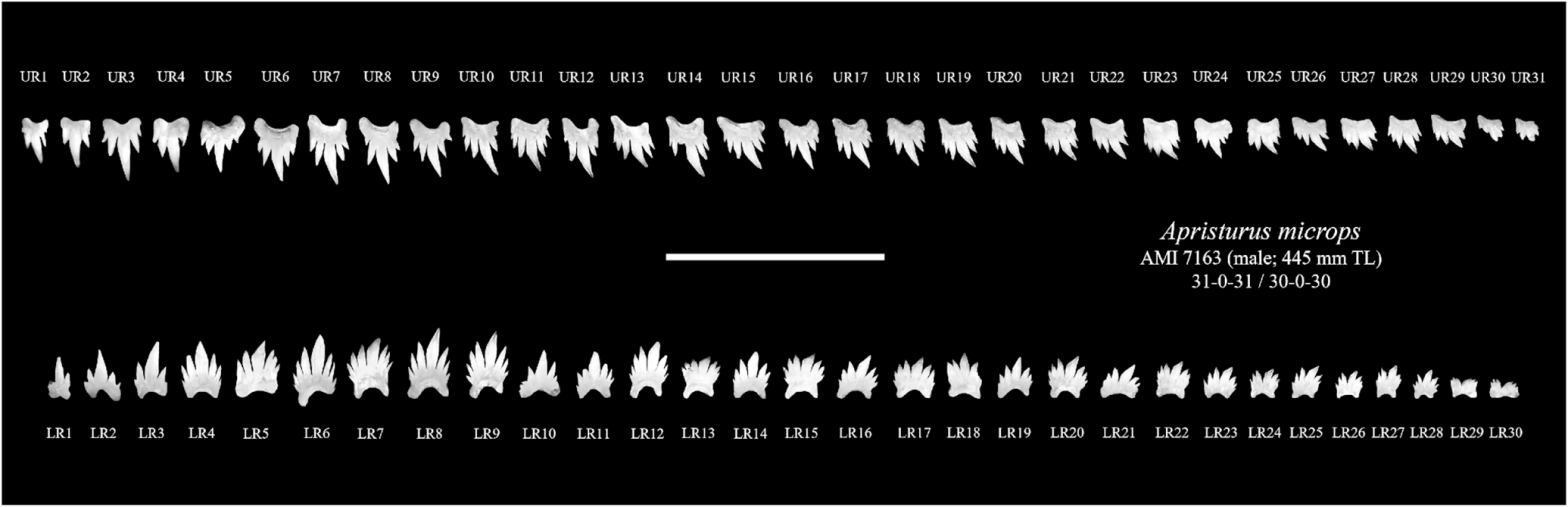
Dentition of the right upper and lower jaw of *Apristurus microps* specimen AMI 7163 (male; 445 mm TL). The image shows the correct position of the teeth from a labial view. Scale bar is 5 mm.

**FIGURE 12:**
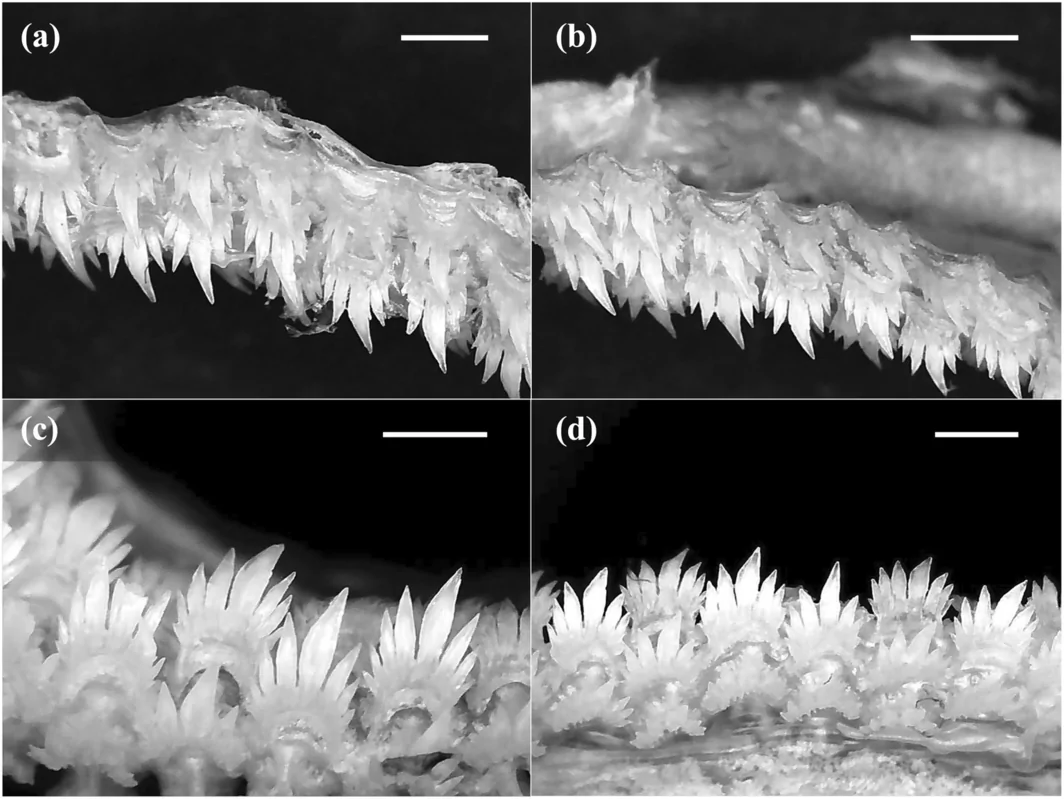
Dentition of *Apristurus microps* specimen AMI 7163 (male; 445 mm TL) in situ: upper jaw, UR7–13 (a), upper jaw, UR16–22 (b), lower jaw LR5–9 (c) and lower jaw, LR12–17 (d). Scale bar is 1 mm.

**FIGURE 13:**
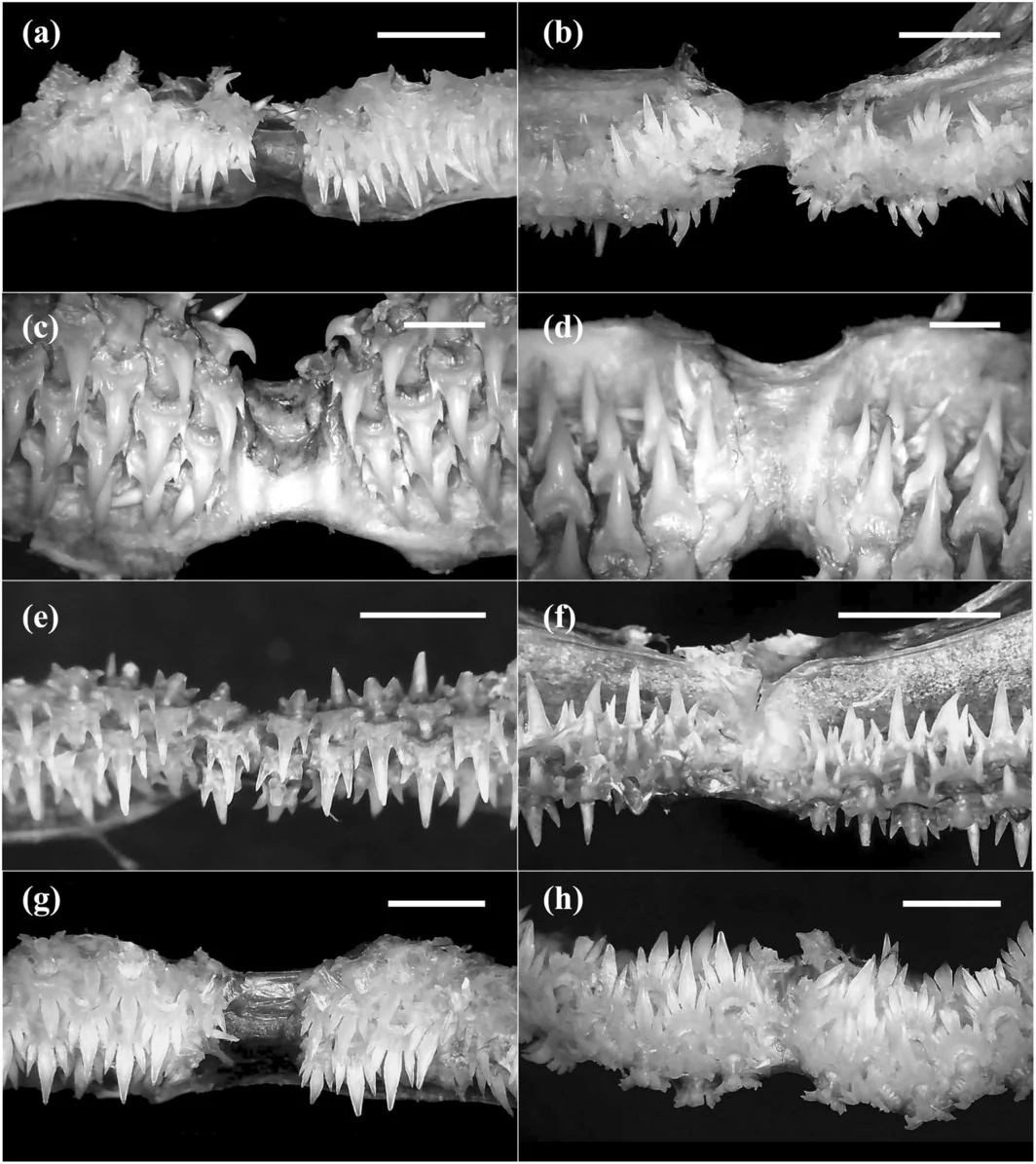
Naked space at the symphysis in four Northeast Atlantic *Apristurus* species: *Apristurus aphyodes* specimen AAP 7783 (a, b; female; 394 mm TL), *Apristurus manis* specimen DE200304‐001 (c, d; male; 720 mm TL), *Apristurus melanoasper* specimen AME 1067 (e, f; female 588 mm TL) and *Apristurus microps* specimen AMI 7163 (g, h; male; 445 mm TL). Scale bar is 2 mm.

**FIGURE 14:**
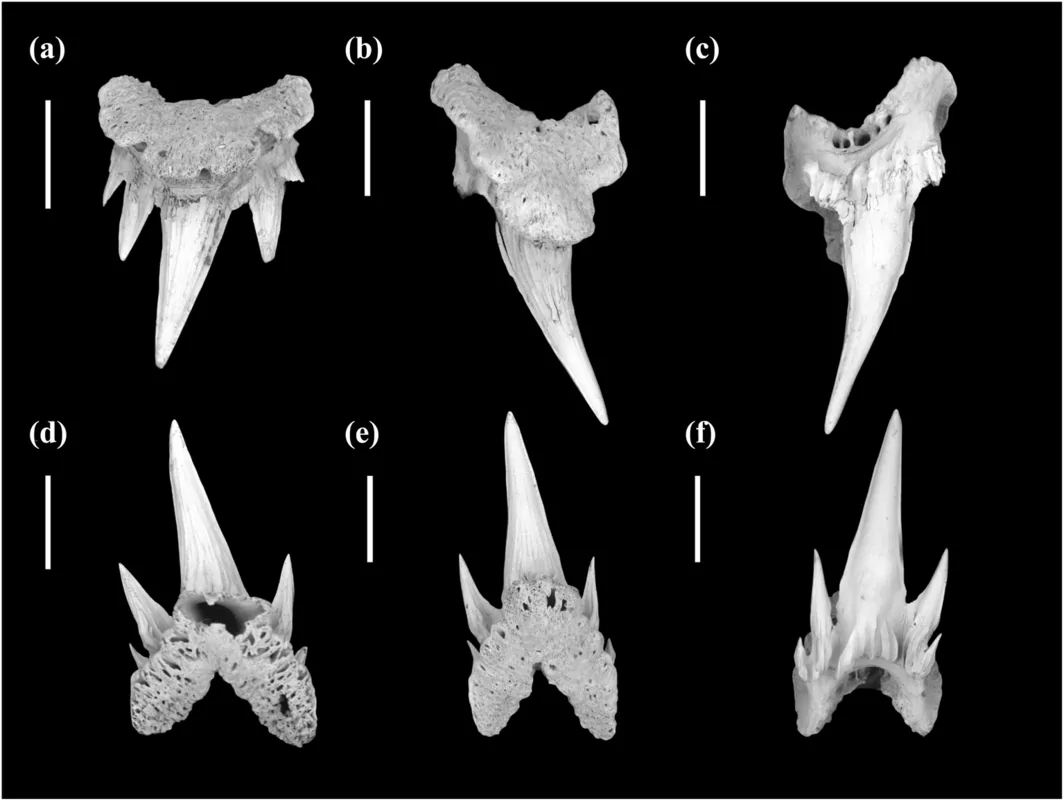
Isolated teeth of *Apristurus aphyodes* specimen AAP 4010 (male; 538 mm TL). (a) Right upper lateral tooth (lingual view); (b, c) Left upper anterior tooth (lingual and labial view); (d) Right lower lateral tooth (lingual view); (e, f) Right lower lateral tooth (lingual and labial view). Scale bar is 500 μm.

**FIGURE 15:**
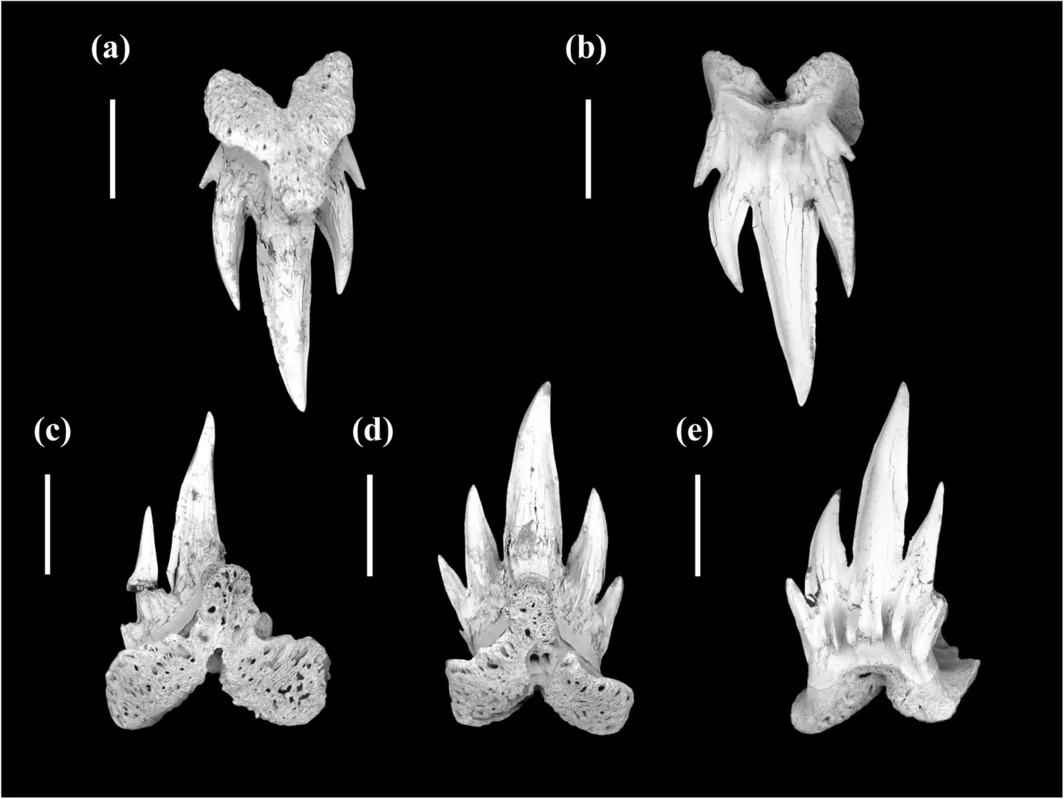
Isolated teeth of *Apristurus aphyodes* specimen AAP 7782 (female; 533 mm TL). (a, b) Right upper anterior tooth (lingual and labial view); (c) Left lower lateral tooth (lingual view); (d, e) Right lower anterior tooth (lingual and labial view). Scale bar is 500 μm.

**FIGURE 16:**
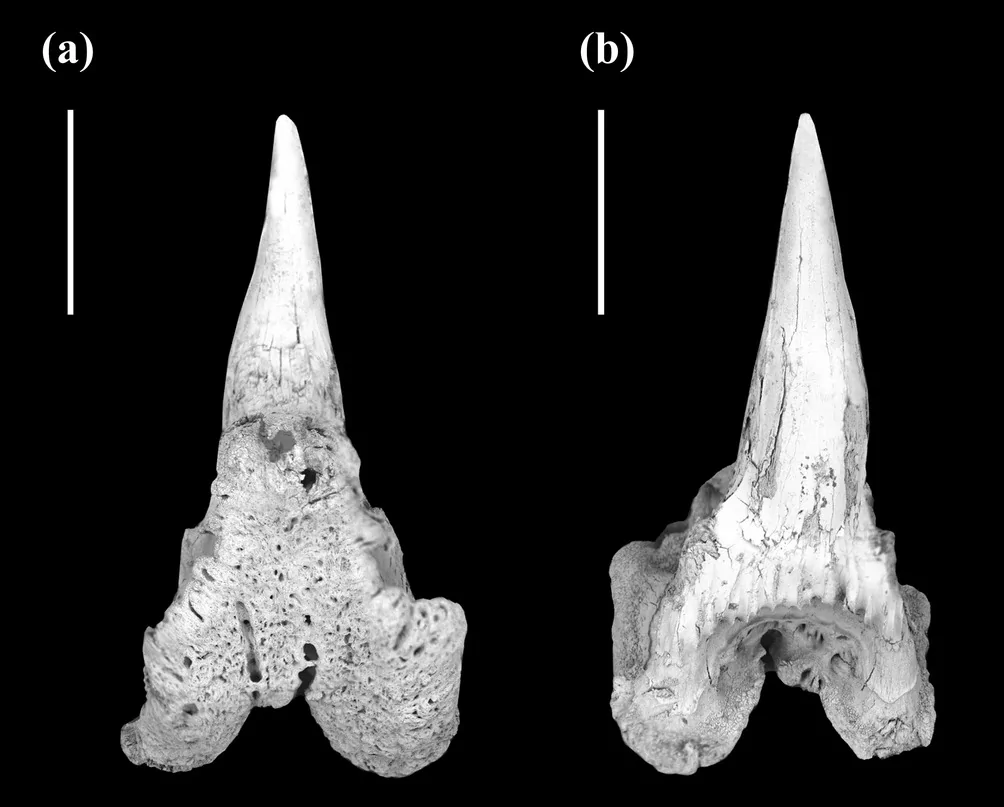
Isolated teeth of *Apristurus manis* specimen DE200304‐001 (male; 720 mm TL). (a, b) Left lower anterior tooth (lingual and labial view). Scale bar is 500 μm.

**FIGURE 17:**
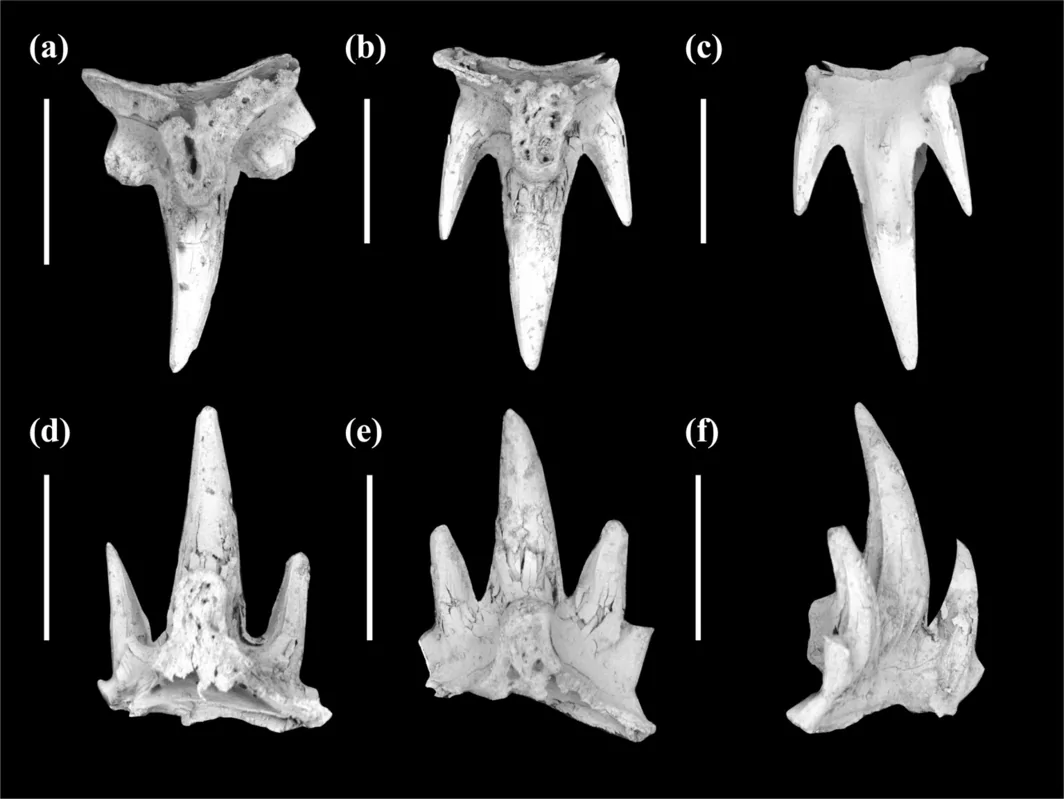
Isolated teeth of *Apristurus melanoasper* specimen AME 1067 (female; 588 mm TL). (a) Right upper anterior tooth (lingual view); (b, c) Right upper lateral tooth (lingual and labial view); (d) Right lower lateral tooth (lingual view); (e, f) Right lower lateral tooth (lingual and labial view). Scale bar is 500 μm.

**FIGURE 18:**
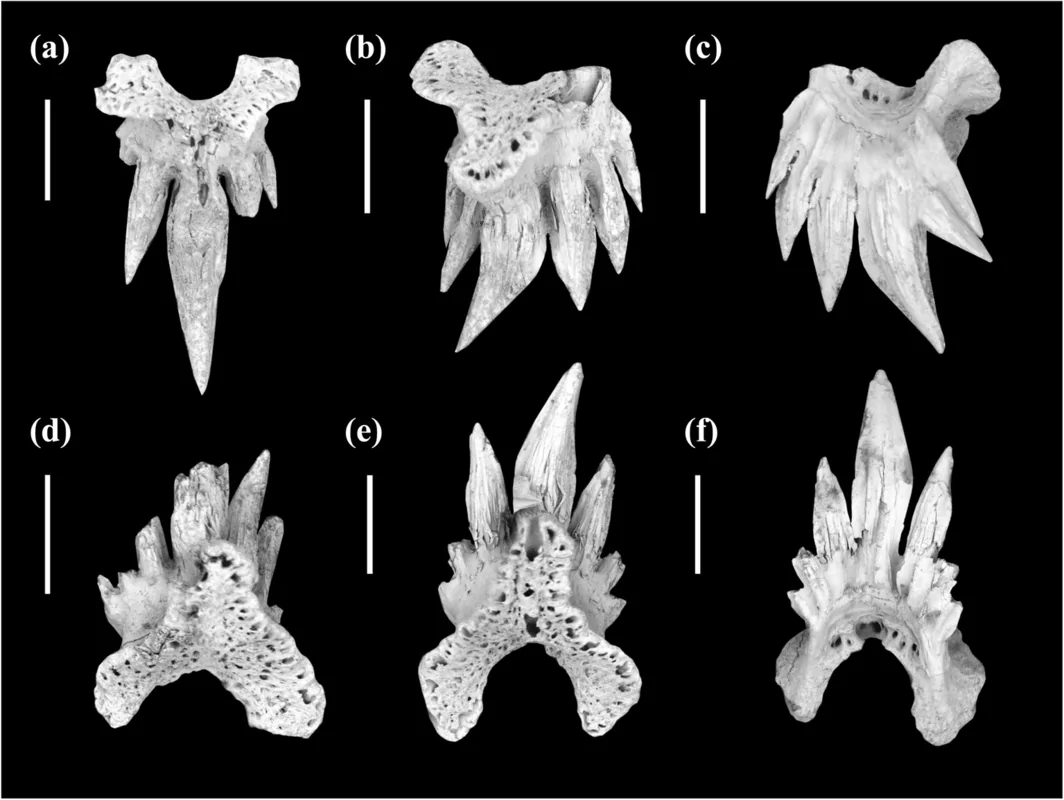
Isolated teeth of *Apristurus microps* specimen AMI 7163 (male; 445 mm TL). (a) Right upper anterior tooth (lingual view); (b, c) Right upper posterior tooth (lingual and labial view); (d) Left lower lateral tooth (labial view); (e, f) Left lower lateral tooth (lingual and labial view). Scale bar is 500 μm.

The selection of imaging equipment and settings was optimised to ensure consistent and comparable image quality across specimens. For composite figures comprising multiple images (Figures 6, 7, 8, 9, 10 and 11), a uniform magnification level was maintained within each photo. The photographs were processed using GIMP image‐editing software, version 3.0.4 (https://www.gimp.org/).

The nomenclature regarding dentition and tooth morphology follows Herman et al. (1990), Compagno (2001) and Zanini and Soares (2025). The following abbreviations are used to indicate the tooth positions in the respective jaw quadrant (labial view): UR/UL/US (upper jaw right/left/symphysis) and LR/LL/LS (lower jaw right/left/symphysis). For example, the abbreviation UR5 refers to the 5th tooth in the right upper jaw (counted from the symphysis from a labial view).

## 3 RESULTS AND DISCUSSION

### 3.1 Dentition of *Apristurus aphyodes* (*spongiceps* species group)

Material examined: See Table 1.

*Dentition*. The dentition of both upper and lower jaws exhibits weak dignathic heterodonty, consisting of small, pointed, conical teeth arranged in multiple, independently sub‐arranged rows (Figure 2). The teeth are multicuspid, with a prominent principal cusp flanked by smaller lateral cusplets. In the upper jaw, anterior teeth possess an erect and nearly symmetrical principal cusp, which becomes progressively more inclined in lateral positions. In males, the curvature of posterior teeth is particularly pronounced, with the tooth tip extending beyond the crown base. In the lower jaw, the principal cusps are straight and show no inclination in anterior and lateral positions; inclination occurs only posteriorly. The cutting edge of the principal cusp is smooth and unserrated. It is nearly straight in anterior teeth, with curvature increasing laterally, especially in the upper jaw. Anterior teeth often display slight basal thickening of the central cusp.

Flanking the principal cusp, the mesial and distal cutting edges each typically bear one or two slender, pointed triangular cusplets in males, and two to three in females. In males, anterior cusplets do not exceed one‐fourth of the total crown height, while in posterior teeth they may reach up to one‐third. In females, cusplets are more numerous and taller relative to crown height: Even in anterior teeth, they reach half the height of the principal cusp, increasing distally until, in posterior teeth, they nearly equal the principal cusp in height. Posterior cusplets also exhibit slight inclination, mirroring that of the principal cusp (Figures 6 and 7).

Both jaws contain five to six consecutive tooth rows, with a naked space present in the symphyseal region in both sexes. Maximum tooth height is 2.5 mm (LR6) in a male specimen of 504 mm total length (TL), and 2.0 mm (LR1) in a female specimen of 533 mm TL.

*Morphology of isolated teeth*. The labial surface of the principal cusp and cusplets is flat to weakly convex and bears weak, straight striae on the tooth crown. These striae are more pronounced at the basal portion of the crown and gradually diminish toward the apex. On the cusplets, the striae are slightly more pronounced than on the principal cusp and range from straight to weakly sigmoidal in pattern. Areas of the tooth surface not traversed by striae are smooth.

The lingual surface of the tooth is convex and is uniformly ornamented with straight to weakly sigmoidal striae, which are significantly more numerous and more pronounced than those on the labial surface. Approximately eight or more striae are present on the principal cusp, extending continuously from the base of the crown to the apex. On the cusplets, the striae are comparable in development to those on the labial surface.

The root comprises two elongate, narrow lobes forming a basal angle. A prominent central foramen is absent on the lingual face of the root in most specimens; instead, smaller foramina are distributed across the entire lingual root surface. The size of these foramina increases apically (Figures 14 and 15).

*Sexual dimorphism*. *A. aphyodes* exhibits pronounced sexual dimorphism in the form of gynandric heterodonty. The dentition of males is characterised by larger, more pointed, straighter and more erect teeth, which possess a single (or in lateral and posterior positions up to two) lateral cusplets on each edge of the principal cusp. In contrast, the teeth of mature females are more compact and comb‐shaped, featuring two or three lateral cusplets on each side, and are notably less erect, particularly in posterior positions (Figures 6 and 7). This observation is supported by the records of Nakaya and Stehmann (1998).

*Tooth Formula*. The dental formula of *A. aphyodes* was established by Nakaya and Stehmann (1998) as 56–68/49–64, based on the holotype and paratypes; a range that has since been adopted in subsequent literature (e.g., Ebert et al., 2021; Ebert & Stehmann, 2013). Examination of the specimens in this study yielded a formula of 56–70/50–57. This extends the previously documented range to 56–70/49–64. Additionally, there are indications of an ontogenetic increase in tooth count from juvenile to adult stages, although this observation warrants verification through a larger sample size.

### 3.2 Dentition of *Apristurus laurussonii* (*brunneus* species group)

Material examined: None.

*Dentition*. The teeth of this species possess a relatively long, slender and elongate principal cusp, which is more strongly developed in the upper jaw, representing a condition of weak dignathic heterodonty (Herman et al., 1990; Zanini & Soares, 2025). The dentition is organised in an independent sub‐arrangement with the principal cusp inclined toward the distal region and typically flanked by one, two or, in lateral teeth, three similarly slender and elongate cusplets on each side (Herman et al., 1990; Zanini & Soares, 2025). According to Springer (1979), most teeth within the jaw are tricuspid. The principal cusp possesses a smooth, unserrated cutting edge that is nearly straight in upper jaw anterior and lateral teeth (Herman et al., 1990). Its curvature increases distally and is more pronounced in the lower jaw, where it is also present in lateral teeth (Herman et al., 1990).

The cusplets adjacent to the principal cusp attain at least half its height, often exceeding this proportion in lower lateral teeth (Herman et al., 1990), where the number of cusplets is also generally greater compared to anterior teeth (Iglésias & Nakaya, 2004). In lower jaw posterior teeth, the principal cusp is so strongly curved that it is almost less high than the adjacent cusplets (Herman et al., 1990).

Both jaws contain more than four consecutive tooth rows, with a small naked space present in the symphyseal region of the upper jaw in males (Bourdon, 2005; Ebert et al., 2021). Maximum height of anterior teeth measures about 1.5 mm in a male specimen with 640 mm TL (Bourdon, 2005) and nearly 1.0 mm in a female specimen with 680 mm TL (Herman et al., 1990).

*Morphology of isolated teeth*. The description of the dental morphology of *A. laurussonii* follows the characterisation by Herman et al. (1990) based on a female specimen with 680 mm TL, which was supported by comprehensive SEM images:

The labial face of the principal cusp and cusplets is flat to weakly convex, displaying well‐developed, predominantly regular striae that occasionally appear slightly sigmoidal. Striae density is highest on anterior teeth (up to six on the principal cusp) and decreases laterally (to two on lower lateral teeth). Striae on cusplets are fainter. Ectodermal pits occur at the crown base.

In contrast, the lingual face is strongly convex with somewhat less distinct striae. These run regularly from the crown base toward the apex, maintaining a density of about six on the principal cusp and fewer on cusplets. Ectodermal pits are absent.

The root consists of two elongated, narrow lobes that form a basal angle. A prominent central foramen is present on the lingual root face, forming a line with two to four foramina on each lobe near the crown and along the ridge. Additional foramina are randomly scattered across the basal face.

*Sexual dimorphism*. Reports on sexual dimorphism in this shark species are somewhat inconsistent. Most earlier authors, including Cadenat & Blache (1981), Compagno (1984) and Springer, 1979, describe an absence of significant gynandric heterodonty, particularly concerning mouth and tooth size. In contrast, Iglésias and Nakaya (2004) identified morphological distinctions, noting that females possess a wider mouth and a greater number of lateral cusplets. This observation is consistent with more recent findings of weak gynandric heterodonty by Zanini and Soares (2025), suggesting a subtle yet present sexual dimorphism in dentition. Such an interpretation is further supported by the comparative analysis of male and female *A. laurussonii* dentitions presented by Bourdon (2005) and Herman et al. (1990).

*Tooth Formula*. The dental formula for this species was established by Iglésias and Nakaya (2004) as 54–102/43–106 (based on an examination of 29 specimens) and has since been adopted in subsequent standard works (Ebert et al., 2021; Ebert & Dando, 2021; Ebert & Dando, 2024). Furthermore, the study by Iglésias and Nakaya (2004) documented an ontogenetic increase in the number of tooth rows with age.

### 3.3 Dentition of *Apristurus manis* (*spongiceps* species group)

Material examined: See Table 1.

*Dentition*. The dentition of both upper and lower jaws displays weak dignathic heterodonty, characterised by small, pointed, conical teeth organised in an independent sub‐arrangement in multiple rows in both jaws (Figure 3). The teeth are multicuspid, characterised by a dominant principal cusp flanked by smaller lateral cusplets. This morphology conforms to the description by Springer (1979), who originally described the species as *Parmaturus manis*. Anteriorly, the principal cusp is erect and nearly symmetrical, becoming progressively more inclined in distal positions. This inclination is more pronounced in the upper jaw than in the lower. In the most posterior teeth, the cusp tip extends almost beyond the crown base. The principal cusp presents a smooth, unserrated cutting edge that is nearly straight in anterior teeth, with curvature increasing laterally, especially in upper jaw teeth. Anterior teeth frequently exhibit slight basal thickening of the central cusp.

Adjacent to the principal cusp, the mesial and distal cutting edges typically bear one or two, rarely three, slender, pointed and triangular cusplets. In anterior teeth, these cusplets do not exceed one‐third of the total crown height, whereas in posterior teeth, they may attain up to half the maximum height. Posterior cusplets also display a slight inclination, mirroring that of the principal cusp (Figure 8).

Both jaws contain five to six consecutive tooth rows, with a naked space present in the symphyseal region. Maximum tooth height measures 3.5 mm (LR4) in a male specimen with 720 mm TL.

*Morphology of isolated teeth*. The labial aspect of the main cusp is markedly convex and exhibits faint, linear ornamentation in the form of striae across the crown surface. These striae are most distinct near the crown base, where they locally broaden into shallow furrows and progressively fade toward the apical region. Portions of the crown lacking striae are smooth, and a continuous, unserrated cutting edge extends along the crown margin.

The lingual side of the tooth is convex and consistently decorated with straight striae that remain subtle overall but are distinctly more developed than those on the labial surface. On the principal cusp, approximately 10 or more striae are present and extend uninterrupted from the basal margin of the crown to its apex.

The root is composed of two slender, elongate lobes that meet at a basal angle. A well‐developed central foramen is present on the lingual root face, accompanied by numerous smaller foramina dispersed across the entire lingual surface. These foramina increase in diameter toward the apical direction. In addition, basal grooves may occur on the labial surface of the root, extending from the basal margin toward the larger foramina (Figure 16).

*Sexual dimorphism*. The species has previously been described as exhibiting pronounced sexual dimorphism in the form of gynandric heterodonty, with adult males possessing enlarged mouths and teeth (Compagno, 1984; Ebert & Stehmann, 2013). This observation can neither be confirmed nor refuted in the current study due to a lack of suitable specimens.

*Tooth Formula*. The tooth count for the primary specimen is 61/56 in the upper/lower jaws, respectively, with additional specimens exhibiting counts of 60/55 and 59/51. While Springer (1979), based on his examination of type specimens, documented a tooth formula of 59/52, Ebert et al. (2021) described a broader range of 59/52–62. Based on the combined evidence, the tooth formula for *A. manis* can be updated to 59–61/51–62. [Correction added on 07 July 2026, after first online publication: The preceding dental formula has been corrected from “59–61/–62” to “59–61/51–62”.]

### 3.4 Dentition of *Apristurus melanoasper* (*brunneus* species group)

Material examined: See Table 1.

*Dentition*. The dentition of this species displays weak dignathic heterodonty, characterised by small, pointed, multicuspid teeth arranged in multiple, independent sub‐arranged rows (Figure 4). A long, robust principal cusp forms the centre of each tooth, flanked by smaller, slender lateral cusplets. In the upper jaw, the principal cusp is more strongly developed. Anterior teeth feature an erect and nearly symmetrical principal cusp, which becomes progressively more inclined in lateral positions. In contrast, the principal cusps in the lower jaw are straight in anterior and lateral positions, showing curvature only posteriorly. The cutting edge is smooth and unserrated, being nearly straight in anterior teeth with curvature increasing laterally, a feature more evident in the upper jaw.

The mesial and distal cutting edges typically bear one or two pointed and erect, triangular cusplets. In the upper jaw, the anterior teeth exhibit a single pair of cusplets. In the lateral teeth, the cusplet distribution is mostly asymmetrical, with two cusplets located on the mesial edge and one cusplet on the distal edge of the teeth. However, there are also occasionally lateral teeth with one or two pairs of symmetrically arranged cusplets. The upper posterior teeth again display only a single pair of cusplets. In the lower jaw, the first anterior teeth possess a single pair of cusplets, whereas teeth in the lateral and posterior regions normally exhibit two pairs of cusplets each in females and one pair of cusplets in males, although up to two pairs may occur in anterolateral positions. In both the upper and lower jaws, the cusplets reach approximately half the height of the principal cusp. This ratio increases slightly with increasing posterior position of the teeth (Figures 9 and 10).

Both jaws contain four to six consecutive tooth rows, with a naked space present in the symphyseal region of the lower jaw, but not in the upper jaw. Maximum tooth height is ca. 0.7 mm (UR7 and LR2–LR5) in a male specimen of 312 mm TL and 1.2 mm (UR5 and LR5) in a female specimen of 588 mm TL.

*Morphology of isolated teeth*. In labial view, the principal cusp is strongly convex and displays only extremely weak, thread‐like striation on the crown. These striae originate near the crown base and taper apically, without forming the deep basal furrows observed in *A. aphyodes* or *A. manis*. The lateral cusplets show the same type of faint, straight striation, with no appreciable difference in intensity relative to the principal cusp. Apart from this delicate ornamentation, the labial crown surface is nearly featureless and smooth.

The lingual side of the tooth is evenly convex and likewise bears fine striae, which are comparable to those on the labial surface and are also present on the cusplets. In contrast to other species of *Apristurus*, the striation is relatively sparse, with only approximately six weak striae developed on the principal cusp.

The root consists of two narrow, elongate lobes converging at a basal angle. On the lingual face of the root, a central foramen is developed in some specimens, while numerous smaller foramina are scattered across the surface. These foramina show a gradual increase in size toward the apical region (Figure 17).

*Sexual dimorphism*. Weak gynandric heterodonty is evident in *A. melanoasper*. Males possess taller, more erect teeth with fewer lateral cusplets, typically a single pair in the upper jaw and one to two pairs in the lower jaw. In comparison, the dentition of females is characterised by shorter teeth bearing a greater number of cusplets, generally one to two pairs in both the upper and lower jaws (Iglésias & Nakaya, 2004; Nakaya et al., 2008). The male and female specimens examined here correspond to this description, although it appears to be somewhat less pronounced in smaller individuals.

*Tooth Formula*. In the original description of *A. melanoasper*, based on Northeast and Northwest Atlantic specimens, Iglésias and Nakaya (2004) reported a dental formula of 59–93/58–97. Subsequently, the species was documented from Australian, New Zealand, New Caledonian, Indian Ocean and eastern South Atlantic waters. Examination of these specimens by Nakaya et al. (2008) yielded a tooth formula of 63–86/57–88. The tooth formulae of the specimens examined in the present study fall within these two reported ranges and a combined formula of 59–93/57–97 can be established for *A. melanoasper*. An ontogenetic increase in the number of tooth rows with age is documented for this species (Iglésias & Nakaya, 2004), which is supported by the specimens examined here.

### 3.5 Dentition of *Apristurus microps* (*spongiceps* species group)

Material examined: See Table 1.

*Dentition*. The dentition of both upper and lower jaws displays weak dignathic heterodonty, composed of small, pointed and fanned teeth organised in multiple, independently sub‐arranged rows (Figure 5). The teeth are multicuspid, featuring a principal cusp flanked by smaller lateral cusplets. The principal cusp is markedly thickened centrally and exhibits a characteristic lanceolate shape in both jaws, with a smooth and unserrated cutting edge. In anterior teeth, the principal cusp is nearly symmetrical; a distal inclination develops laterally, which is more pronounced in the upper jaw than in the lower jaw.

The mesial and distal cutting edges adjacent to the principal cusp typically bear two to four broad, elongated and lanceolate cusplets. These cusplets attain approximately half the crown height in anterior teeth, increasing distally to about two‐thirds of the total height. Mesial cusplets incline toward the symphysis, whereas distal cusplets are oriented toward the commissure. Anterior teeth generally possess two pairs of lateral cusplets, lateral teeth commonly have three and posterior teeth may bear up to four. Cusplets in the lower jaw are generally more numerous and pronounced than those in the upper jaw (Figures 11 and 12).

Both jaws contain four to six consecutive tooth rows, with a naked space present in the symphyseal region of the upper jaw, but not in the lower jaw. Maximum tooth height measures 1.5 mm (positions UR7 and LR8) in a male specimen of 445 mm TL.

The dental morphology of the *A. microps* specimen examined in this study differs markedly from previous descriptions by Smith (1961) as well as from the illustrations provided by Ebert and Dando (2021) and Ebert et al. (2021). In those accounts, the teeth are depicted as upright, slender and conical, with the principal cusp flanked by only a single lateral cusplet on each side. More recent observations by Ebert and Dando (2024) indicate the presence of one to two pairs of lateral cusplets. By contrast, the teeth of the specimen analysed here are more compact, distinctly fan‐shaped and bear a substantially higher number of lateral cusplets (up to four pairs). This morphological deviation may be attributable to several factors. Ontogenetic change is the most plausible explanation, as the present specimen was a juvenile individual (maturity scale A/1 according to Stehmann, 2002). Alternatively, sexual dimorphism or intraspecific variation could account for the differences, particularly given the species' patchy distribution, which may have led to morphological divergence between isolated populations. The available data for this shark species are extremely limited, and a definitive assessment would require a larger sample size, encompassing both male and female specimens across different stages of sexual maturity from various locations in the Atlantic Ocean.

*Morphology of isolated teeth*. When observed from the labial side, the principal cusp appears markedly arched and is characterised by conspicuous ornamentation in the form of striae. The striae extend continuously from the basal margin of the crown toward the apex, becoming progressively finer apically. Near the crown base, they broaden into deep furrows, particularly at the bases of the cusplets. On the cusplets, the striation is equally well developed or locally more pronounced than on the main cusp. Aside from this ornamentation, the crown surface is largely smooth.

The lingual surface of the tooth is convex and likewise bears well‐developed striae, although these are slightly weaker than those on the labial side. As on the labial surface, the striae extend from the basal crown to the apex and expand basally into broader furrows. On the cusplets, the degree of striation matches that observed on the principal cusp.

The root is composed of two slender, strongly elongated lobes that converge at a basal angle. On the lingual root face, a prominent central foramen is present in the majority of specimens, accompanied by numerous smaller foramina distributed across the surface. Toward the apical margin of the root, these foramina increase in size and form a distinct, band‐like arrangement (Figure 18).

*Sexual dimorphism*. Gynandric heterodonty has not been documented in *A. microps*, due to the limited data available for this species. As only a single juvenile male specimen was examined in this study, no conclusions regarding sexual dimorphism could be drawn. Further research with a more comprehensive sample, including mature individuals of both sexes, is needed to investigate this possibility.

*Tooth Formula*. The established dental formula for the species is 62–66/59–62 (Ebert et al., 2021). The specimen examined here, with a count of 62/60, falls within this range.

### 3.6 Differentiation of the dentition of the *Apristurus* species from the Northeast Atlantic Ocean

While the dental morphology of Northeast Atlantic *Apristurus* species is generally similar, it nevertheless exhibits discernible interspecific variation. Teeth are uniformly small and multicuspid with one principal cusp across all species, though the number and shape of lateral cusplets varies considerably between species, sexes and even within the teeth of individuals (Table 2). A pronounced divergence in tooth size between clades is particularly evident (especially in males), since teeth in *A. spongiceps* clade specimens are generally larger and more erect than those of the *A. brunneus* clade. Due to the regional focus of this study, this conclusion would need to be investigated more thoroughly for other *Apristurus* species worldwide.

**TABLE 2:** Overview of the dentition characteristics in *Apristurus* species of the Northeast Atlantic Ocean (N/A = Not available/not known).

Sex	*Apristurus laurussonii*		*Apristurus melanoasper*		*Apristurus aphyodes*		*Apristurus manis*		*Apristurus microps*	
	Male	Female	Male	Female	Male	Female	Male	Female	Male	Female
Gynandric heterodonty	Weak		Weak		Strong		Strong		N/A	
Dignathic heterodonty	Weak	Weak	Weak	Weak	Weak	Weak	Weak	N/A	Weak	N/A
Lateral cusplets in upper anterior teeth^a^	2	4	2	2	2	2–4	2–3	N/A	2–4	N/A
Lateral cusplets in upper lateral teeth^a^	2–3	4–5	2–4	2–4	2–4	4–6	2–3	N/A	4–6	N/A
Lateral cusplets in upper posterior teeth^a^	2–4?	2–4	2	2	2–4	3–5	4	N/A	4–5	N/A
Lateral cusplets in lower anterior teeth^a^	2–4	4	2–4	2–4	2	4	0–2	N/A	2–6	N/A
Lateral cusplets in lower lateral teeth^a^	2–4	6	2–4	4	2–4	4–6	2	N/A	6–8	N/A
Lateral cusplets in lower posterior teeth^a^	2–4?	4–6	2–3	3–4	2–4	4–5	2–4	N/A	6–8	N/A
Naked space at symphysis (upper)	Yes (small)	N/A	No	No	Yes	Yes	Yes	N/A	Yes	N/A
Naked space at symphysis (lower)	No	N/A	Yes	Yes	Yes	Yes	Yes	N/A	No	N/A
Tooth count (upper)	54–102		59–93		56–70		59–61		62–66	
Tooth count (lower)	43–106		57–97		49–64		51–62		59–62	
Ontogenetic increase of the tooth count	Yes		Yes		Yes		N/A		N/A	
References	Bourdon (2005); Herman et al. (1990); Iglésias and Nakaya (2004)		Present study; Iglésias and Nakaya (2004); Nakaya et al. (2008)		Present study; Ebert and Stehmann (2013)		Present study; Ebert et al. (2021)		Present study; Ebert and Dando (2024), Ebert et al. (2021)	

^a^ Refers to the total number of cusplets without the principal cups (= the cusplets on the mesial and distal edge of the principal cusp combined).

Sexual dimorphism expressed as gynandric heterodonty is present to varying degrees in all examined *Apristurus* species. The primary differences between male and female dentition concern tooth height and the number of lateral cusplets. In all specimens assessed, females exhibited less erect teeth and a greater number of lateral cusplets than males (Table 2). Species of the *A. spongiceps* clade show a more pronounced expression of this dimorphism, whereas it is weak or possibly absent in some members of the *A. brunneus* clade. Further support for this pattern is provided by records of other *Apristurus* species worldwide (Compagno, 1984; Springer, 1979).

Moreover, a naked (toothless) space at the symphyseal region was consistently observed across all examined jaws within the *A. brunneus* and *spongiceps* clade (Figure 13). Consistent with this observation, Springer (1979) reported this characteristic for *Apristurus* species in general but did not restrict it to particular species or sexes, and suggested that it may be related to the degree of relaxation of the symphyseal ligaments. Subsequently, this feature has been reported in several other *Apristurus* species across different clades, such as *A. nakayai* (*A. brunneus* clade), where it was suggested as a potential sexually dimorphic trait (Ng et al., 2023). This hypothesis was also examined for the species described herein, but can be refuted at least for *A. aphyodes* and *A. melanoasper*, as both males and females possess the gap. In the male individual of *A. manis*, the naked space is present in both jaws, whereas in male *A. laurussonii* and *microps*, it is confined to the upper jaw. In *A. melanoasper*, it is limited to the lower jaw and in male *A. laurussonii*, the toothless gap appears to be relatively small compared to the other species; however, more specimens are required to support this finding. For the remaining females, no conclusions regarding this feature can be drawn due to limited sample sizes. At present, the causes of the naked space remain speculative, as does the question of whether it truly represents a species‐ or sex‐specific trait.

Additionally, there are indications for an ontogenetic change in the number of tooth rows in three Northeast Atlantic species of *Apristurus* (*A. aphyodes*, *A. laurussonii* and *A. melanoasper*), with the number of tooth rows increasing progressively with age. This phenomenon has been documented in numerous other shark species from various families (Purdy & Francis, 2007; Reif, 1976; Seifert, 2025; Tomita et al., 2017) and is of particular relevance, since standard taxonomic literature often provides a dental formula as a diagnostic character for species identification (Ebert et al., 2021). In species with a high number of tooth rows, such as *Apristurus* spp., in which this number is additionally subject to ontogenetic increase, the reliability of the current tooth formulae as an identifying character cannot always be ensured. This is further evidenced by discrepancies between the results of the present study and previously published data. It is therefore of particular importance to document characteristics of dentition more comprehensively in future publications, especially in first descriptions, in order to reliably use dental formulae as identification features for *Apristurus* species.

Finally, it should be noted that representatives of the *A. spongiceps* clade possess on average a larger mouth and jaw relative to the TL than representatives of the *A. brunneus* clade. This difference is reflected in measurements of the DUJP, DLJP, as well as the outer measurements of the dried jaw. The ratios of DUJP, DLJP and jaw size to total body length are higher in the *A. spongiceps* clade specimens than in representatives of the *A. brunneus* clade (Table 3). This pattern is consistent with previously published results for *Apristurus* species of both clades worldwide (Compagno, 1984; Ebert et al., 2021; Gilbert, 1905; Springer, 1979).

**TABLE 3:** Dried upper jaw perimeter (DUJP), dried lower jaw perimeter (DLJP) and jaw size relative to the total length (TL) in *Apristurus* species of the Northeast Atlantic Ocean (N/A = Not available/not known;/= Placeholder for summarised data per species).

Species	Collection number	TL in mm	DUJP in mm	DLJP in mm	(DUJP + DLJP)/TL in %	Mean (species) in %	Standard deviation (species) in %	Jaw width in mm	Jaw height in mm	(Width + height)/TL in %	Mean (species) in %	Standard deviation (species) in %
*A. aphyodes*	AAP 1063	460	55	44	21.5	21.5	2.1	60	53	24.6	24.2	2.4
	AAP 4010	538	70	55	23.2	/	/	83	60	26.6	/	/
	AAP 4018	309	36	26	20.1	/	/	37	34	23.0	/	/
	AAP 7234	504	69	52	24.0	/	/	78	59	27.2	/	/
	AAP 7780	465	46	35	17.4	/	/	52	40	19.8	/	/
	AAP 7781	408	56	40	23.5	/	/	60	49	26.7	/	/
	AAP 7782	533	61	44	19.7	/	/	72	54	23.6	/	/
	AAP 7783	394	51	37	22.3	/	/	48	40	22.3	/	/
*A. laurussonii* ^a^	N/A	640	63	54	18.3	18.3	0.0	68	60	20.0	20.0	0.0
*A. manis*	DE200304‐001	720	108	83	26.5	23.5	2.1	109	105	29.7	26.6	2.3
	DE200304‐002	720	87	72	22.1	/	/	95	80	24.3	/	/
	DE200304‐006	700	88	66	22.0	/	/	95	85	25.7	/	/
*A. melanoasper*	AME 1066	312	36	26	19.9	16.3	3.5	35	31	21.2	17.3	3.8
	AME 1067	688	49	39	12.8	/	/	49	44	13.5	/	/
*A. microps*	AMI 7163	445	61	50	24.9	24.9	0.0	68	53	27.2	27.2	0.0

^a^ The measurements for *Apristurus laurussonii* were taken from the photographed specimen published by Bourdon (2005).

## 4 CONCLUSION

This study provides the first comprehensive comparative analysis of the dentition and tooth morphology for all *Apristurus* species within the Northeast Atlantic Ocean. It establishes a detailed reference framework of dental characteristics, including heterodonty patterns, sexual dimorphism and tooth formulae. For the first time, pronounced gynandric heterodonty in *A. aphyodes* has been visually documented, coinciding with an ontogenetic increase of the tooth rows. Moreover, this study provides signs of an ontogenetic change in dental morphology within *A. microps* and further supports this finding for *A. melanoasper*. The detailed tooth morphology of *A. aphyodes*, *A. laurussonii*, *A. manis* and *A. melanoasper* is presented for the first time in a comparative framework, providing an important basis for future identification.

The improved identifiability of different *Apristurus* species can directly lead to more accurate species‐specific distribution data, refining biogeographic models and conservation assessments for these cryptic deep‐sea sharks. Furthermore, this detailed atlas of extant dental morphology serves as an essential reference for paleontological research. It provides comparative material for identifying isolated fossil and subfossil shark teeth, enabling more precise reconstructions of past faunal assemblages and environmental conditions in the Atlantic basin.

While this study consolidates data for the region, limitations such as the uneven availability of specimens across species and sexes are acknowledged. Future research should focus on expanding sample sizes to confirm intra‐ and interspecific characteristics in order to strengthen the informative value of tooth morphology when used for species identification. Extending this morphological approach to similar species would also be valuable for understanding global diversity and evolution of other deep‐sea sharks.

## AUTHOR CONTRIBUTIONS

Jesco Seifert: conceptualisation, methodology, investigation, data recording, specimen preparation, writing – original draft, writing – review and editing, visualisation. Daniel M. Moore: conceptualisation, methodology, specimen collection, writing – original draft, writing – review and editing.

## CONFLICT OF INTEREST STATEMENT

The authors declare that they have no known competing financial interests or personal relationships that could have appeared to influence the work reported in this paper.

## Data Availability

The data that support the findings of this study are available from the authors upon reasonable request.
